# Determination of the prevalence of African trypanosome species in indigenous dogs of Mambwe district, eastern Zambia, by loop-mediated isothermal amplification

**DOI:** 10.1186/1756-3305-7-19

**Published:** 2014-01-10

**Authors:** Malimba Lisulo, Chihiro Sugimoto, Kiichi Kajino, Kyouko Hayashida, Macarthy Mudenda, Ladslav Moonga, Joseph Ndebe, Selestine Nzala, Boniface Namangala

**Affiliations:** 1 Department of Biomedical Sciences, School of Medicine, University of Zambia, P.O. Box 50110, Lusaka, Zambia; 2 Research Centre for Zoonosis Control, Hokkaido University, Kita-Ku, Sapporo 001-0020, Japan; 3 Department of Paraclinical Studies, School of Veterinary Medicine, University of Zambia, P.O. Box 32379, Lusaka, Zambia; 4 Department of Disease Control, School of Veterinary Medicine, University of Zambia, P.O. Box 32379, Lusaka, Zambia; 5 Department of Community Medicine, School of Medicine, University of Zambia, P.O. Box 50110, Lusaka, Zambia

**Keywords:** CAT, HAT, Indigenous dogs, LAMP, Trypanosomes, Mambwe district, Zambia

## Abstract

**Background:**

Dogs have been implicated to serve as links for parasite exchange between livestock and humans and remain an important source of emerging and re-emerging diseases including trypanosome infections. Yet, canine African trypanosomosis (CAT), particularly in indigenous dogs (mongrel breed) remains under- reported in literature. This study evaluated the performance of loop-mediated isothermal amplification (LAMP) in detecting trypanosomes in blood from indigenous dogs of tsetse-infested Mambwe district in eastern Zambia.

**Methods:**

A cross sectional survey of CAT was conducted within 5 chiefdoms (Msoro, Kakumbi, Munkanya, Nsefu, Malama) of Mambwe district, eastern Zambia, during October 2012. Blood samples from 237 indigenous hunting dogs were collected and screened by microscopy and LAMP.

**Results:**

Of the 237 dogs screened for CAT, 14 tested positive by microscopy (5.9%; 95% CI: 2.9 – 8.9%), all of which also tested positive by LAMP. In addition, LAMP detected 6 additional CAT cases, bringing the total cases detected by LAMP to 20 (8.4%; 95% CI: 4.9 – 12.0%). Irrespective of the detection method used, CAT was only recorded from 3 chiefdoms (Munkanya, Nsefu, Malama) out of the 5. According to LAMP, these infections were caused by *Trypanosoma congolense*, *Trypanosoma brucei brucei* and the zoonotic *Trypanosoma brucei rhodesiense*. Although these CAT cases generally did not manifest clinical illness, an association was observed between infection with *Trypanosoma brucei* subspecies and occurrence of corneal opacity.

**Conclusions:**

This communication reports for the first time the occurrence of CAT in indigenous Zambian dogs. Our study indicates that LAMP is a potential diagnostic tool for trypanosome detection in animals. LAMP was more sensitive than microscopy and was further capable of distinguishing the closely related *T. b. brucei* and *T. b. rhodesiense*. In view of the sporadic cases of re-emerging HAT being reported within the Luangwa valley, detection of the human serum resistant associated (SRA) gene in trypanosomes from mongrels is intriguing and indicative of the risk of contracting HAT by local communities and tourists in Mambwe district. Consequently, there is a need for continuous trypanosome surveillances in animals, humans and tsetse flies using sensitive and specific tests such as LAMP.

## Background

In almost all societies, dogs are widely utilised and offer several benefits to humans, with the main one being security [1]. However, from a public health perspective, dogs have been sources of zoonotic parasites [2,3]. As such, dogs have served as a link for parasite exchange among livestock, wildlife and humans, and remain an important source of emerging and re-emerging infectious diseases [4,5].

In tsetse-infested sub-Saharan African countries, pathogenic protozoan trypanosome species are transmitted to a wide range of susceptible mammalian hosts, including dogs, through infective tsetse fly (*Glossina*) bites when taking blood meals [6,7]. Specifically, dogs are affected by *Trypanosoma congolense*, *Trypanosoma evansi* and *Trypanosoma brucei* subspecies [8-10], causing canine African trypanosomosis (CAT). In exotic breeds of dogs, *T. brucei* subspecies tend to cause acute CAT [5] while infections caused by *T. congolense* appear to be more chronic [5,9,11]. According to Abenga *et al.*[6], indigenous dog breeds in tsetse-infested regions of sub-Saharan Africa seem to be trypanotolerant. Although such dogs get infected with trypanosomes, they either exhibit subclinical signs or may not exhibit any overt clinical signs of the disease at all. Consequently, such dogs may also act as sources of infection to other domesticated animals and, more importantly, those with the human-infective *T. brucei rhodesiense* and *T. brucei gambiense* may serve as a source of infection for humans [5,8,11-13].

In our recent communication on CAT in exotic dog breeds [5], we reported for the first time in Zambia the occurrence of CAT caused by *T. congolense* and *T. brucei* subspecies. Through the use of LAMP, we had observed that some dogs were infected with the human-infective *T. b. rhodesiense*, suggesting their potential to act as sources of HAT. Two of the dogs had contracted CAT from the tsetse-infested Mambwe district in eastern Zambia, with one of them testing positive for *T. b. rhodesiense* infection.

To further address the paucity of data on CAT, we extended our investigations in this study to a relatively larger sample size of indigenous dogs, within the vicinity of South Luangwa National Park (SLNP) in the 5 chiefdoms of Mambwe district, namely Msoro, Kakumbi, Munkanya, Nsefu and Malama. We further evaluated the performance of the trypanosome species-specific LAMP, using parasite DNA obtained from the indigenous dog blood samples, against the gold standard microscopy.

## Methods

### Study site and design

During the month of October 2012, a cross sectional survey of CAT involving a total of 237 indigenous dogs was conducted in 47 villages within 5 chiefdoms (Msoro, Kakumbi, Nsefu, Munkanya, Malama) of Mambwe district. The mongrels comprised 128 males and 109 females, most of which were hunting dogs aged between 3 months and 16 years. Mambwe district is situated in the eastern province of Zambia along the Luangwa valley, which supports a high density of tsetse flies and is a historic HAT focus. It lies within Lupande game management area (GMA) between latitudes 10° and 15° South and longitudes 30° and 33° East [14] adjacent to SLNP and is a popular tourist destination.

### Sample collection and microscopy

Blood samples were conveniently collected from indigenous dogs whose owners consented to participate in the survey. Each participating dog was clinically examined and its body condition scored as described by [15]. Datum specific for each dog was captured on record sheets. About 2 ml of blood was drawn from the cephalic vein of each dog into heparinised capillary tubes, packed cell volume (PCV) values determined and Giemsa-stained thin blood smears from each dog examined as described [16]. In addition, about 200 μl of each blood sample was placed on a labeled FTA® Elute card (Whatman FTA® Elute Cards, Whatman, UK) for DNA extraction according to the manufacturer’s suggested protocol. All the participating dogs in this study were freely vaccinated against rabies.

### DNA extraction and loop-mediated isothermal amplification

DNA was extracted from dog blood samples and used for the LAMP assay as described by Namangala *et al*. [5], using specific primers targeting the 18S rRNA gene of *T. congolense* (CON2-LAMP) [17], the repetitive insertion mobile element (RIME) gene of the *Trypanozoon* subgenus group (RIME-LAMP) [18] and the human serum resistance-associated (SRA) gene uniquely expressed by *T. b. rhodesiense* (SRA-LAMP) [19], respectively. All RIME-LAMP positive samples were screened for *T. b. rhodesiense* using SRA-LAMP. Samples that were RIME-LAMP positive and SRA-LAMP negative were considered to be *T. b. brucei*.

### Data analysis

The captured data were entered, stored and statistically analysed using STATA version 11.0. Mean PCV values in trypanosome infected and non-infected dogs were compared using the one-way analysis of variance. The Fisher’s Exact test was used to determine whether an association existed between the outcome variable (microscopy and LAMP results) and categorical variables under consideration (risk factors and clinical signs). Fisher’s Exact test was used in the place of the chi squared test due to the small frequencies in some of the categories tested. Binary logistic regression was used to determine the true predictors of being positive for CAT by microscopy and LAMP. P values <0.05 were considered statistically significant and only variables with significant Fishers exact test p-values were further analysed using univariable logistic regression. Based on their potential biological significance, a number of factors that included; chiefdom, age, body condition, vision, mucous membranes and the practice of hunting were entered into a multivariable logistic regression model. The multivariable model was constructed by first including all variables that passed the initial screening and then dropping variables manually in a backwards elimination procedure based on the likelihood ratio test. Only variables that were still significant at 5% level in the likelihood ratio test were retained.

### Ethical clearance

Approval to conduct this study was granted by the University of Zambia Biomedical Research Ethics Committee under reference number 020-07-12. Informed consent was sought from dog owners to participate in the survey and collect blood from their dogs.

## Results

### Clinical appearance of the examined indigenous dogs

**Figure 1:**
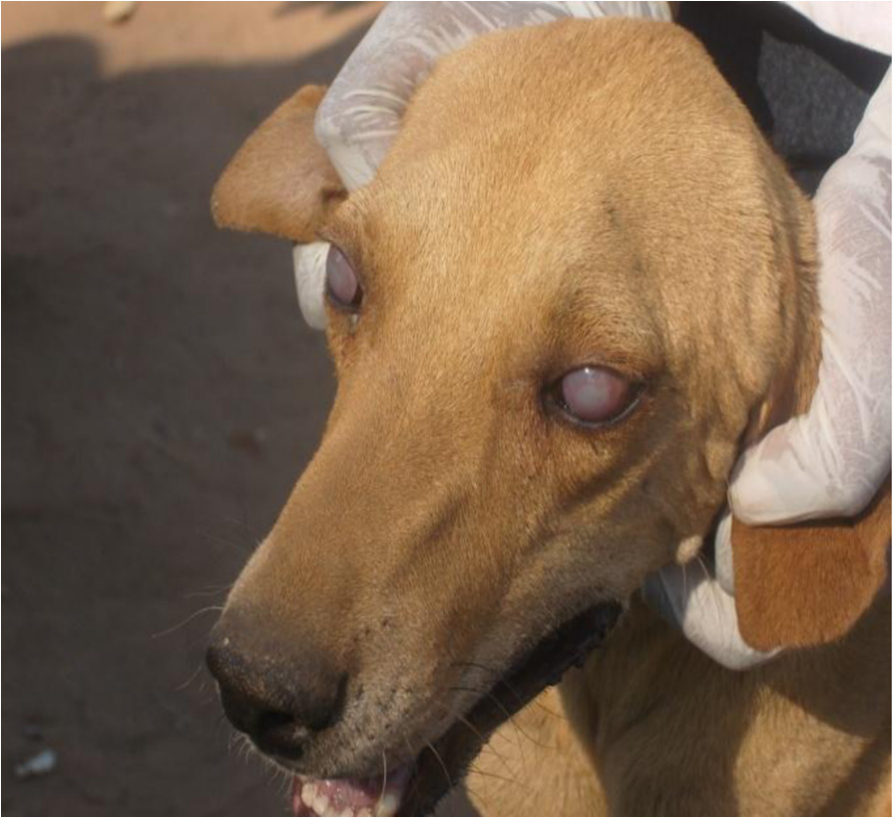
Clinical examination of an indigenous dog showing evidence of bilaterial corneal opacity.

Physical examinations revealed that a total of 31 (13.1%; 95% CI: 8.8 – 17.4%) out of the 237 dogs were emaciated, another 22 (9.3%; 95% CI: 5.6 – 13.0%) had pale mucous membranes. As observed by Bwalya *et al.*[20], most of the examined dogs in this study were also seen passing tapeworms and round worms in their stools during sampling. Furthermore, 55 dogs (23.2%; 95% CI: 17.8 – 28.6%) were infested with ecto-parasites, mainly ticks of *Rhipicephalus* species, predominantly found in the ears and inter digital spaces. About 24 dogs (10.1%; 95% CI: 6.3 – 14.0%) had enlarged superficial lymph nodes while 5 (2.1%; 95% CI: 0.3 – 4.0%) exhibited bilateral corneal opacity (Figure 1).

### Detection of African trypanosomes by microscopy and LAMP

According to microscopy, 14 out of 237 mongrels (5.9%; 95% CI: 2.9 – 8.9%), were found to be infected with trypanosomes. All those 14 cases were also found to be positive for trypanosome infection by LAMP. Moreover, LAMP detected 6 additional CAT cases, bringing the total CAT cases detected by LAMP to 20 (8.4%; 95% CI: 4.9 – 12.0%), (Table 1). According to multivariable logistic regression analysis, the risk factors that seemed to influence the prevalence of CAT in indigenous dogs included location/chiefdom, (Odds ratio (OR) 6; 95% CI: 2.0 – 16.4; p = 0.002), vision (OR 18; 95% CI: 1.2 – 267.3; p = 0.037), age (OR 8.0; 95% CI: 1.0 – 61.1; p = 0.049) and illegal hunting (OR 2; 95% CI: 1.0 – 10.0; p = 0.049. Thus, although no CAT cases were recorded from Msoro and Kakumbi chiefdoms, Munkanya, Malama and Nsefu chiefdoms each recorded CAT prevalences of 18.0%, 16.7% and 8.8% by LAMP, respectively (Table 1). Adult dogs that were involved in hunting were more likely to acquire CAT than young dogs. In addition, although not statistically significant (p = 0.147), dogs that were positive for CAT tended to have lower PCVs (29 ± 9.4%; 95% CI: 23 – 32%) than those that tested negative (32 ± 8.7%; 95% CI: 31 – 33%).

**Table 1:** Prevalence of trypanosome species in indigenous dogs of Mambwe district, eastern Zambia, by LAMP

**Chiefdom**	**N° of dogs**	**Mean PCV (%) of infected dogs**	**Infected with**					**Overall prevalence of infection**
			*T. c*	*T. b. b*	*T. b. r*	*T. c + T. b. b*	*T. c + T. b. r*	
**Kakumbi**	36	N/A	0 (0.0%) (0.0 – 0.0%)	0 (0.0%) (0.0 – 0.0%)	0 (0.0%) (0.0 – 0.0%)	0 (0.0%) (0.0 – 0.0%)	0 (0.0%) (0.0 – 0.0%)	0.0% (0.0 – 0.0%)
**Malama**	30	30 ± 14.1	0 (0.0%) (0.0 – 0.0%)	0 (0.0%) (0.0 – 0.0%)	3 (10.0%) (0.0 – 21.4%)	0 (0.0%) (0.0 – 0.0%)	2 (6.7%) (0.0 – 16.1%)	16.7% (2.5 – 30.8%)
**Msoro**	53	N/A	0 (0.0%) (0.0 – 0.0%)	0 (0.0%) (0.0 – 0.0%)	0 (0.0%) (0.0 – 0.0%)	0 (0.0%) (0.0 – 0.0%)	0 (0.0%) (0.0 – 0.0%)	0.0% (0.0 – 0.0%)
**Munkanya**	50	27.7 ± 8.4	2 (4.0%) (0.0 – 9.6%)	2 (4.0%) (0.0 – 9.6%)	1 (2.0%) (0.0 – 6.0%)	2 (4.0%) (0.0 – 9.6%)	2 (4.0%) (0.0 – 9.6%)	18.0% (7.0 – 29.0%)
**Nsefu**	68	32 ± 5.7	1 (1.5%) (0.0 – 4.4%)	2 (2.9%) (0.0 – 7.1%)	2 (2.9%) (0.0 – 7.1%)	0 (0.0%) (0.0 – 0.0%)	1 (1.5%) (0.0 – 4.4%)	8.8% (1.9 – 15.7%)
**Total**	**237**	**29 ± 9.4**	**3 (1.3%)** (0.0 - 2.7%)	**4 (1.7%)** (0.0 – 3.3%)	**6 (2.5%)** (0.5 – 4.5%)	**2 (0.8%)** (0.0 – 2.0%)	**5 (2.1%)** (0.3 – 4.0%)	**8.4%** (4.9 – 12.0%)

N/A: Not applicable; *T. c: Trypanosoma congolense; T. b. b: Trypanosoma brucei brucei; T. b. r: Trypanosoma brucei rhodesiense.*

According to LAMP, 13 mongrels (5.5%; 95% CI: 0.7 – 10.6%) were monolytically infected, 3 with *T. congolense*, 4 with *T. b. brucei* and 6 with *T. b. rhodesiense*, respectively. The other 7 dogs (2.9%; 95% CI: 0.6 – 5.9%) were co-infected, 2 with *T. congolense* and *T. b. brucei* and 5 with *T. congolense* and *T. b. rhodesiense* (Table 1). Among the infected dogs, 4 had corneal opacity (2 monolytic infections with *T. b. rhodesiense*; 1 monolytic infection with *T. b. brucei*; 1 co-infection with *T. b. rhodesiense* and *T. congolense*). Only 1 dog with corneal opacity tested negative for CAT. Analysis of data using Fisher’s exact test revealed that CAT was significantly associated with corneal opacity. Accordingly, dogs with corneal opacity were 18 times more likely to harbour *T. brucei* subspecies than dogs with normal vision (OR 18; 95% CI: 1.2 – 267.3; p = 0.037).

The diagnostic accuracy of LAMP (index) was determined against microscopy (reference standard). As shown in Table 2, LAMP was found to be more sensitive and specific, and had a positive likelihood ratio (LR) of 37 and a negative LR of 0, suggesting that its use in CAT diagnosis could improve the management of this disease in endemic areas.

**Table 2:** Diagnostic accuracy of LAMP and microscopy

	**Microscopy**		
	**CAT present**	**CAT absent**	**Total**
LAMP-positive	True positive (TP) = 14	False positive (FP) = 6	**TP + FP = 20**
LAMP-negative	False negative (FN) = 0	True negative(TN) = 217	**TN + FN = 217**
**Total**	**TP + FN = 14**	**TN + FP = 223**	**237**

Note:

LAMP Sensitivity = TP / (TP + FN) = 100%.

LAMP Specificity = TN / (TN + FP) = 97.3%.

LAMP Positive predictive value (PPV) = TP / (TP + FP) = 70%.

LAMP Negative predictive value (NPV) = TN / (TN + FN) = 100%.

LAMP Positive likelihood ratio (LR+) = sensitivity / (1 – specificity) = 37.

LAMP Negative likelihood ratio (LR-) = (1 – sensitivity) / specificity = 0.

## Discussion

The present study evaluated the performance of LAMP against the gold standard microscopy to detect trypanosomes in the blood collected from indigenous Mambwe mongrels in eastern Zambia. Whereas microscopy detected 14 CAT cases (5.9%), LAMP detected 20 CAT cases (8.4%) out of the 237 mongrels that were available for sampling in the present study, including all the 14 parasitologically positive cases, suggesting that LAMP is a reliable test. LAMP was found to have a positive likelihood ratio (LR) of 37 and a negative LR of 0. According to Florokowski [21], a positive LR > 10 and a negative LR < 0.1 can exert highly significant changes in probability that is capable of altering clinical management. Our findings therefore show that the use of LAMP in CAT diagnosis could improve the management of this disease in endemic areas. Furthermore, LAMP was not only able to distinguish different trypanosome species such as *T. congolense* and *T. brucei*, but was also able to distinguish between closely related *T. b. brucei* and *T. b. rhodesiense*.

According to LAMP, about 35% of the CAT cases were co-infections, supporting the notion that many CAT infections in the field are caused by more than one trypanosome species [13]. Based on experimental findings in co-infected dogs, *T. brucei* subspecies are thought to dominate and interfere with *T. congolense* in establishing parasitaemia and subsequently their pathogenic effects [13]. In further agreement with previous reports [8,13], the presence of either *T. b. brucei* or *T. b. rhodesiense* infections in dogs was characterized by relatively higher parasitaemia and in some cases bilateral corneal opacity, unlike monolytic infections with *T. congolense*. As previously reported [8,11,13,22,23], the loss of vision in dogs due to corneal opacity seems to be associated with monolytic or mixed infections with *T. brucei* subspecies. Dogs with corneal opacity were 18 times more likely to be CAT infected than the dogs with normal vision (OR 18; 95% CI: 1.2 – 267.3; p = 0.037). Importantly, however, no association with corneal opacity was observed in monolytic infections with *T. congolense* in the present study. This may not be surprising as members of *T. brucei* subspecies, unlike *T. congolense*, can traverse the vascular tissue and cause damage to various extra-vascular tissues, including the eyes [5,8].

The present findings are consistent with those of other studies and further support the idea that LAMP tends to be more sensitive than microscopy [5,18,24,25]. LAMP has the advantage over other sensitive and specific molecular techniques such as polymerase chain reaction (PCR) in that it is simpler, more rapid and cheaper to perform, as it only requires a heating device for incubation and may therefore be performed even in the field [26,27]. Since our laboratory extracts DNA by simply boiling the Elute FTA disks at 95°C for 30 minutes (Whatman FTA® Elute Cards, Whatman, UK), we are in the process of preparing LAMP reagents that would conveniently enable us to conduct the assay in the field. Furthermore, the LAMP assay shows high tolerance to biological products such that DNA extraction may not be necessary [28]. Thus LAMP may be more practical for routine diagnosis of CAT and other neglected tropical protozoan diseases in resource-limited communities of sub-Saharan Africa where such infections are endemic [25].

Our findings show that location (chiefdom), age and illegal hunting were significant risk factors that predisposed indigenous dogs to CAT. Out of the 5 chiefdoms surveyed, CAT was only detected in Munkanya (18.0%), Malama (16.7%) and Nsefu (8.8%) mainly among adult hunting dogs while no CAT cases were reported in Kakumbi and Msoro. The human-infective *T. b. rhodesiense* was detected in each of the 3 chiefdoms where CAT was reported. According to the predictive epidemiological theory, where the non-human-infective *T. b. brucei* and the human-infective *T. b. rhodesiense* subspecies co-exist, the prevalence of the latter should exceed that of the former [29]. In contrast, however, most field reports suggest the opposite to be true and show that *T. b. brucei* predominates in all domestic livestock [30]. Our data seems to be in conformity with the predictive epidemiological theory. In particular, the unusually high prevalence of the human-infective *T. b. rhodesiense* circulating in dogs from Malama chiefdom compared to the non-human-infective *T. b. brucei* is intriguing. Similarly, although the overall prevalence of the non-human-infective *T. b. brucei* reported by Hamill *et al.*[30] in northern Tanzania was higher than that of the human-infective *T. b. rhodesiense*, Arumeru district recorded unusually higher prevalence of the human-infective *T. b. rhodesiense* circulating in pigs. These data should be used to trigger a “One Health” approach towards HAT control through disease intervention in livestock [30]. On the other hand, however, the prevalence of the non-human-infective *T. b. brucei* in our study may be under-estimated considering the fact that our LAMP system is unable to identify dogs co-infected with *T. b. rhodesiense* and *T. b. brucei*. The difference in CAT distribution among chiefdoms may be as a result of (i) the proximity to SLNP, availability and abundance of tsetse flies and other haematophagous arthropods, (ii) the occurrence of larger domestic livestock which may be more preferable for tsetse feeding than the smaller mongrels [14], (iii) the frequency of hunting by dogs, hence their chance of being bitten by tsetse flies, and (iv) the heavy presence of Zambia wildlife authority (ZAWA) personnel that monitor acts of illegal hunting in Kakumbi and Msoro chiefdoms. Thus, the fact that no domestic animals other than dogs are kept in Malama may partially explain the higher CAT prevalence, whereas the presence of livestock in wildlife zones [14] and the availability of veterinary services for dogs mainly in Kakumbi and Msoro may explain its absence.

In conformity with a recent report of CAT in exotic dogs [5], this research reports two main trypanosome species causing CAT in indigenous dogs, i.e. *T. congolense* and *T. brucei* subspecies. However, whereas *T. brucei* subspecies reportedly cause acute CAT in exotic dog breeds [5,8], no acute CAT was observed among indigenous Mambwe dogs. In fact, most of the CAT cases in this research did not manifest clinical disease other than corneal opacity in some cases. These data suggest that indigenous dogs in tsetse-infested regions such as Mambwe district may be relatively tolerant to trypanosome infections [5,6,8]. Ideally, those dogs should have shown more serious clinical signs considering the fact that most of them were malnourished, generally had poor body condition scores and never received veterinary services. Nonetheless, the majority of those dogs were either asymptomatic or only exhibited mild signs, with no cases of acute CAT. As such, they could serve as reservoirs of infection for other domestic animals and humans [5,22].

The detection of the SRA gene in trypanosomes isolated from 11 indigenous dogs in this study is a source of public health concern in view of the close relationship between dogs and humans. Matete [8] postulated that “sporadic and very low prevalence of human-infective trypanosomes in dogs closely reflects disease occurrence in humans”. It is noteworthy that Anderson *et al*. [31] recently reported SRA positive trypanosomes in an African buffalo in Nyamaluma, within Malama chiefdom where we detected the same gene in trypanosomes from 5 indigenous hunting dogs. These findings indicate the risk of contracting HAT by the local communities, ZAWA officials and tourists. Indeed, unpublished confirmed sporadic cases of HAT have previously been reported in Mambwe district [32], with the most recent case involving a 21 year-old man from Munkanya chiefdom. Moreover, in 2008 alone, between the months of March and July, about 12 HAT cases involving ZAWA officials were reported in the Luangwa valley [33]. Furthermore, at least 6 HAT cases have been reported among tourists visiting SLNP since 2010 [34-36]. Collectively, these data suggest that HAT is re-emerging in the old foci within the Luangwa river valley [26,34-36].

## Conclusions

Despite the many benefits indigenous dogs may offer to the local communities, our study findings show that dogs are potential links for tryapanosome exchange between livestock and humans. For instance, the detection of the SRA gene in trypanosomes from dogs from Zambia’s Mambwe district indicates the risk of humans contracting HAT. Our study suggests that LAMP has a higher sensitivity and specificity than microscopy and is hence a more reliable test in detecting trypanosomes. In order to facilitate effective prevention and control measures of trypanosome infections, there is a need for continuous surveillance of the disease in tsetse-infested regions such as Mambwe district, using user-friendly and yet sensitive and specific tests such as LAMP. It is particularly important to sensitize the local community of the potential dangers of keeping dogs that are just left to scavenge without receiving any veterinary services in such a prohibited place like the GMA. Such dogs may harbour several other zoonoses in addition to *T. b. rhodesiense*, with potential serious implications to human health.

## Abbreviations

CAT: Canine African trypanosomosis; HAT: Human African trypanosomiasis; SLNP: South Luangwa national park; SRA: Serum resistance-associated gene; GMA: Game management area; ZAWA: Zambia wildlife authority; LAMP: Loop-mediated isothermal amplification; RIME: Repetitive insertion mobile element; PCR: Polymerase chain reaction

## Competing interests

The authors declare that they have no competing interests.

## Authors’ contributions

ML helped to conceive the study, participated in its design, obtained funding, collected samples, performed microscopy, purified the DNA from dog blood on FTA elute cards, performed the LAMP Assays, analysed data and drafted the manuscript. MM, LM, and JN were involved in the purification of DNA from dog blood on FTA elute cards and performed the LAMP Assays. SN co-supervised the study and helped in editing the manuscript. BN supervised and helped to conceive the study, participated in its design, collected samples, performed the LAMP Assays and edited the manuscript. SC, KK and HK helped to conceive the study, participated in its design and assisted in obtaining funding. All the authors read and approved the final manuscript.
